# Plasma C1q/TNF-Related Protein-3 (CTRP-3) and High-Mobility Group Box-1 (HMGB-1) Concentrations in Subjects with Prediabetes and Type 2 Diabetes

**DOI:** 10.1155/2016/9438760

**Published:** 2016-09-22

**Authors:** Huili Wei, Hua Qu, Hang Wang, Huacong Deng

**Affiliations:** Department of Endocrinology, The First Affiliated Hospital of Chongqing Medical University, Chongqing 400016, China

## Abstract

*Aims*. To detect the association of C1q/TNF-related protein-3 (CTRP-3) and high-mobility group box-1 (HMGB-1) in subjects with prediabetes (pre-DM) and newly diagnosed type 2 diabetes (nT2DM).* Methods*. 224 eligible participants were included. The 75 g oral glucose tolerance test (OGTT) and several clinical parameters of metabolic disorders and cytokines were measured. All participants were divided into three groups: normal glucose tolerance (NGT, *n* = 62), pre-DM (*n* = 111), and nT2DM group (*n* = 56).* Results*. Plasma CTRP-3 concentrations were significantly lower in subjects with pre-DM and nT2DM than that of the NGT group, while plasma HMGB-1 levels were higher in pre-DM and nT2DM group compared with the NGT group (*P* < 0.05). A multiple linear regression analysis showed both plasma CTRP-3 and HMGB-1 concentrations were independently associated with homeostasis model assessment for insulin resistance (HOMA-IR) and interleukin-6 (IL-6) (*P* < 0.05 for all). Further multiple logistical regression analyses revealed that both plasma CTRP-3 and HMGB-1 levels were significantly associated with pre-DM and nT2DM after adjusting for several confounders (*P* < 0.001 for all).* Conclusions*. Circulating CTRP-3 and HMGB-1 concentrations might be promising biomarkers to predict prediabetes and type 2 diabetes.

## 1. Introduction

Since the dramatic changes in lifestyle worldwide, diabetes has become one of the most common metabolic disorders globally. In 2013, the International Diabetes Federation (IDF) reported 382 million people with diabetes and the number was projected to reach 592 million by 2035 [1]. Of note, another piece of data from IDF also revealed 45.8% or 174.8 million of undiagnosed diabetes in all diabetes cases globally [2]. Prediabetes (pre-DM) is defined as either fasting or postchallenge glycemia, including impaired fasting glucose (IFG), impaired glucose tolerance (IGT), and combined IFG and IGT (impaired glucose regulation, IGR) [3]. As an intermediate state between normal glucose tolerance (NGT) and type 2 diabetes (T2DM), pre-DM is characterized by insulin resistance and impaired insulin secretion [4, 5]. Besides, the chronic low-grade inflammation also contributes to the progression of pre-DM to T2DM [6]. Though most subjects with pre-DM remain clinically asymptomatic, the micro- and macrovascular complications of diabetes already exist in this stage [7]. Recent data have showed that pre-DM is closely associated with poor cardiovascular outcomes [8]. IFG was also found to be correlated with unrecognized myocardial infarctions in patients without fundamental cardiovascular disease [9]. It is estimated that up to 70% of individuals with pre-DM will progress to diabetes at a rate of 5–10% per year [7]. In China, the age-adjusted prevalence of pre-DM is 15.5%, accounting for 148.2 million people with pre-DM [10].

The C1q/tumor necrosis factor- (TNF-) related protein-3 (CTRP-3) belongs to the CTRP family which shares a highly conserved paralog of adiponectin [11]. CTRP-3 is abundantly expressed in the adipose tissue, kidney, uterus, and testis in adult animals [12]. It is well established that CTRP-3 plays an important role in modulating the hepatic glucose and lipid metabolism [13, 14]. In normal C57BL/6 and leptin-deficient ob/ob mice, recombinant CTRP-3 injection displayed a significant antidiabetic effect [13]. Recent clinical studies demonstrate that circulating CTRP-3 is associated with T2DM and obesity [15, 16]. However, conflicting results remain between circulating CTRP-3 and T2DM. Choi et al. [15] reported increased circulating levels of CTRP-3 in subjects with pre-DM and T2DM, while other groups showed lower levels of circulating CTRP-3 in newly diagnosed T2DM patients [16, 17]. Furthermore, CTRP-3 is considered an anti-inflammatory cytokine. In a high-fat fed transgenic mouse model, CTRP-3 mitigated systemic inflammation in the context of obesity and insulin resistance [18]. It has been reported that CTRP-3 could reduce the secretion of interleukin-6 (IL-6) and tumor necrosis factor-*α* (TNF-*α*) by suppressing the nuclear factor-*κ*B (NF-*κ*B) signaling [19].

High-mobility group box-1 (HMGB-1) was first discovered in 1973 [20]. It was originally regarded as an important factor for maintaining the structure and stability of the chromosome [20]. So far, several studies have revealed that HMGB-1 also exerts extracellular functions via HMGB-1 related receptors. For instance, exogenous HMGB-1 can promote the release of proinflammatory cytokines by binding to their receptors such as RAGE, TLR2, and TLR4 [21]. In addition, several studies indicate that HMGB-1 is related to insulin resistance [22], obesity, and T2DM [23]. Wang et al. [23] found plasma HMGB-1 levels were increased in subjects with T2DM and/or obesity. Chen et al. [24] found HMGB-1 was elevated in both T2DM patients and high-glucose cultured SV40 MES 13 cells.

Though previous studies have reported the role of circulating CTRP-3 and HMGB-1 in T2DM and obesity, there is little information concerning the associations of circulating CTRP-3 and HMGB-1 with pre-DM. Pre-DM is a high risk factor of cardiovascular events and plays an important role in the development of diabetes, so studies on biomarkers to predict pre-DM deserve further investigation. In this study, we aim to explore the association of plasma CTRP-3 and HMGB-1 with newly diagnosed pre-DM and T2DM.

## 2. Methods

### 2.1. Study Design and Subjects

A total of 420 subjects (age ranged from 40 to 75) with newly diagnosed T2DM (nT2DM) or pre-DM or healthy controls were recruited from the outpatients of The First Affiliated Hospital of Chongqing Medical University between June 2014 and October 2015. After the 75 g oral glucose tolerance test (OGTT) and routine biochemical check-up, a total of 224 eligible participants were included (the exclusion criteria were presented in Supplemental Figure  1 in Supplementary Material available online at http://dx.doi.org/10.1155/2016/9438760). All participants were divided into normal glucose tolerance group (NGT, *n* = 62), pre-DM group (*n* = 111), and nT2DM group (*n* = 56). The diagnosis of pre-DM and nT2DM was based on the ADA criteria [25]. All enrolled participants have not received any hypoglycemic agents, diet-control, or exercise prior to screening. Present study was in accordance with the Declaration of Helsinki of the World Medical Association and was approved by the Ethics Committee of The First Affiliated Hospital of Chongqing Medical University, Chongqing, China. Written informed consent was obtained from all participants.

### 2.2. Anthropometric, Clinical, and Demographic Measurements

Height, body weight, waist circumference, and hip circumference were measured by standardized methods in all subjects. Blood pressure was measured using an automated electronic device (OMRON Model HEM-725 FUZZY, Omron Company, Dalian, China) three times consecutively after a 5-minute rest; the three readings were averaged for further analyses. Blood samples were collected in the morning (8–10 a.m.) after an overnight fasting (≥8 hours). Plasma samples were obtained by centrifugation at 2000 ×g for 10 minutes at 4°C and were kept at −80°C before analyses. All analyses were performed within one month. Plasma glucose levels were measured using the glucose oxidase method, while HbA1c was measured using the high-performance liquid chromatography (VARIANTTM II and D-10TM Systems, BIO-RAD, USA). Fasting insulin (FINS) was measured using an autoanalyzer (ARCHITECT i2000SR System, Abbott Laboratories, USA). Lipid profiles such as triglyceride (TG), total cholesterol (TC), high-density lipoprotein cholesterol (HDL-C), and low-density lipoprotein cholesterol (LDL-C) were assayed by enzymatic methods, while the liver and kidney functions were determined using a biochemical aut-analyzer (ARCHITECT c16000 System, Abbott Laboratories, IL, USA).

The body mass index (BMI) formula is computed as weight in kilograms divided by height in meters squared. The waist-to-hip ratio (WHR) formula is the waist circumference in centimeters divided by the hip circumference in centimeters. The homeostasis model assessment of insulin resistance (HOMA-IR) = fasting insulin (mU/L) *∗* fasting plasma glucose (mmol/L)/22.5.

### 2.3. Assessment of Plasma CTRP-3, HMGB-1, and IL-6 Levels

Plasma CTRP-3, HMGB-1, and IL-6 levels were determined by commercial ELISA kits according to the manufacturers' instructions (Human ELISA kit, Uscn Life Science Inc., Wuhan, China). The intra-assay coefficients of variation were less than 8%, while the interassay coefficients of variation were less than 10%. All the assays were carried out in duplicate, and no significant cross-reactivity or interference was observed.

### 2.4. Statistical Analyses

The SPSS software (IBM, Armonk, NY, version 19.0) was used for all statistical analyses. Data are presented as means ± standard deviation. Nonnormally distributed parameters including LDL-C and *γ*-GGT were logarithmically transformed before analyses. Comparisons between groups were performed using the analysis of variance (ANOVA). Interrelationships between variables were assessed using a partial correlation analysis by controlling for age and gender. The distributions of plasma CTRP-3 (<395, 395~451, and >455, ng/mL) and HMGB-1 (<4.76, 4.76~5.38, and >5.38, ng/mL) were further divided into tertiles, and the linear trend was estimated by a linear-by-linear association of the chi-square test. A multivariate linear regression was performed to identify the risk factors of plasma CTRP-3 and HMGB-1. Associations among plasma CTRP-3 and HMGB-1 levels and pre-DM and nT2DM were examined using the multiple logistic regression analyses. *P* values < 0.05 were considered statistically significant.

## 3. Results

### 3.1. Characteristics of the Study Subjects

The main characteristics of the subjects are presented in Table 1. Subjects in nT2DM group were older than those of the NGT and pre-DM group (*P* < 0.05). Compared with the NGT group, subjects in the pre-DM and nT2DM group exhibited higher levels of HOMA-IR, WHR, TG, *γ*-GGT, and IL-6, while the levels of HDL-C in the NGT group were higher than those of the pre-DM and nT2DM group (all *P* < 0.05). Besides, subjects in nT2DM group had higher levels of BMI, TC, and LDL-C compared with the pre-DM group (all *P* < 0.05). There were no significant differences in gender, SBP, DBP, ALT, AST, and creatinine distributions among the three groups (*P* > 0.05).

### 3.2. Plasma CTRP-3 and HMGB-1 Levels and Their Associations with Anthropometric and Biochemical Parameters

**Figure 1:**
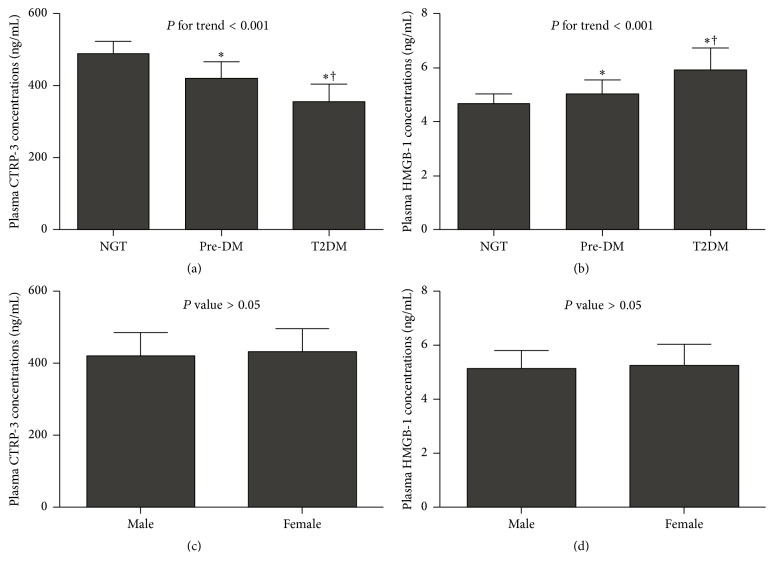
Plasma CTRP-3 (a) and HMGB-1 (b) concentrations in different subgroups and in males and females ((c) and (d)). Data were presented as means ± standard deviation. Differences between multiple groups were tested by analysis of variance (ANOVA) for continuous variables. *P* for trend was estimated by a linear-by-linear association of the chi-square test. ^*∗*^ *P* < 0.05 compared with NGT, ^†^ *P* < 0.05 compared with pre-DM. CTRP-3, C1q/TNF-related protein-3; HMGB-1, high-mobility group box-1; NGT, normal glucose tolerance; pre-DM, prediabetes states (including IFG, impaired fasting glucose; IGT, impaired glucose tolerance; IGR, impaired glucose regulation); T2DM, type 2 diabetes.

As shown in Figures 1(c) and 1(d), there were no significant differences in plasma CTRP-3 ([420.39 ± 64.16] versus [432.14 ± 65.12], ng/mL) and HMGB-1 ([5.13 ± 0.65] versus [5.22 ± 0.78], ng/mL) concentrations between men and women (all *P* > 0.05). Compared with the NGT group (486.548 ± 37.09, ng/mL), subjects displayed a decreased trend of plasma CTRP-3 concentrations in pre-DM (419.649 ± 46.34, ng/mL) and nT2DM group (356.107 ± 49.5, ng/mL) (Figure 1(a), *P* for trend < 0.001). Contrary to CTRP-3, plasma HMGB-1 showed an increased trend across the NGT (4.67 ± 0.37, ng/mL), pre-DM (5.04 ± 0.5, ng/mL), and nT2DM group (5.9 ± 0.81, ng/mL) (Figure 1(b), *P* trend < 0.001).

Then, plasma CTRP-3 levels were divided into tertiles, including the low (<396, ng/mL), medium (398~451, ng/mL), and high group (>455, ng/mL). Similarly, the tertiles of plasma HMGB-1 were divided as the low (<4.76, ng/mL), medium (4.76~5.38, ng/mL), and high group (>5.38, ng/mL). The presence of pre-DM was 53.9, 77.2, and 43.2%, respectively, across the HMGB-1 tertiles (*X*2 = 3.108, *P* trend = 0.026), whereas the presence of nT2DM was 10.5, 12.7, and 54.1%, respectively (*X*2 = 44.07, *P* trend < 0.001). Besides, the proportion of pre-DM was 41, 73.3, and 30.3%, respectively, across the tertiles of CTRP-3 (*X*2 = 16.424, *P* trend < 0.001), while the proportion of nT2DM was 56.4, 14.7, and 6.3%, respectively (*X*2 = 35.607, *P* trend < 0.001).

Partial correlation analyses adjusted by age and gender showed that plasma CTRP-3 was negatively correlated with FPG, 2 h PG, FINS, HOMA-IR, HbA1c, BMI, WHR, TG, LDL-C, and IL-6 and was positively associated with HDL-C (all *P* < 0.05). However, after controlling for age and sex, plasma HMGB-1 was positively correlated with variables including FPG, 2 h PG, FINS, HOMA-IR, HbA1c, BMI, WHR, TG, LDL-C, and IL-6 and was negatively associated with HDL-C (all *P* < 0.05) (Table 2). Further multiple linear regression analyses showed that the plasma CTRP-3 concentrations were independently correlated with HOMA-IR (*β* = −0.247, *P* = 0.009), HbA1c (*β* = −0.437, *P* < 0.001), HDL-C (*β* = 0.144, *P* = 0.048), and IL-6 (*β* = −0.346, *P* < 0.001). In contrast, plasma HMGB-1 concentrations were independently associated with HOMA-IR (*β* = 0.297, *P* < 0.001), IL-6 (*β* = 0.262, *P* = 0.001), and creatine (*β* = 0.118, *P* = 0.017).

Moreover, further multivariate logistic regression analyses revealed that both plasma CTRP-3 and HMGB-1 concentrations were significantly correlated with pre-DM and nT2DM after controlling for several covariates including age, gender, BMI, WHR, blood pressure, TC, TG, LDL-C, HDL-C, and liver and kidney function (*P* < 0.001 for all) (Tables 3 and 4).

## 4. Discussion

Recently, C1q/TNF-related protein-3 (CTRP-3) is considered a secreted hormone that plays a role in the hepatic glucose and lipid metabolism. Several researches have reported the association of circulating CTRP-3 with obesity and T2DM in rodent models and in human [13, 26, 27]. Two studies conducted by Peterson et al. [13, 14] showed that both infusion of recombinant CTRP-3 protein and transgenic overexpression of CTRP-3 in mice were involved in regulating the hepatic gluconeogenesis and lipid metabolism. Recombinant CTRP-3 administration overtly lowered the blood glucose levels in C57BL/6 mice and leptin-deficient obese (ob/ob) mice [13]. In our study, we found plasma CTRP-3 concentrations were significantly lower in subjects with pre-DM and nT2DM compared with the NGT group. A multiple linear regression analysis showed the plasma CTRP-3 levels were independently associated with insulin resistance, HbA1c, and HDL-C. Further multiple logistical analyses indicated that plasma CTRP-3 concentrations were significantly correlated with pre-DM and nT2DM after adjusting for potential confounders. These data manifest that CTRP-3 is an independent and strong predictor for prediabetes and diabetes. However, our study was contrary to Choi et al. [15] who reported higher CTRP-3 concentrations in pre-DM and T2DM.

The potential mechanism of the antiglucose effect of CTRP-3 remains unknown. In mice, increased CTRP-3 may activate the Akt signaling pathway in the liver while simultaneously suppressing the hepatic gluconeogenic gene expression, since CTRP-3 administration suppressed G6Pase and PEPCK (two main gluconeogenic enzymes) by 80% in the murine liver [13]. However, recent study by Wolf et al. [26] found no significant change in the whole body glucose metabolism, insulin sensitivity, and fasting-induced hepatic gluconeogenesis in CTRP-3-deficient mice treated with LFD or HFD. Conflicting results also remain among several clinical studies regarding the association between circulating CTRP-3 and T2DM [15–17].

Furthermore, recent data uncovers the anti-inflammatory properties of CTRP-3 in various models in vivo [28, 29], in vitro [30], and ex vivo [31]. CTRP-3-deficient mice showed remarkably deteriorated inflammatory joint pathology in a collagen-induced rheumatoid arthritis model [28]. In CTRP-3 knock-out mice, high-fat diet suppressed its liver and adipose expression of profibrotic TGF*β*1 and serum TGF*β*1 concentrations, while greatly increasing serum IL-6 levels [26]. Previously, Schmid et al. [29] reported recombinant CTRP-3 administration could attenuate the systemic inflammation in wild-type mice challenged with a sublethal dose of bacterial-derived lipopolysaccharide (LPS). Contrary to this report, Petersen et al. [32] found neither overexpression nor deficiency of CTRP-3 could affect circulating IL-1b, IL-6, or TNF-*α* levels in acute LPS-challenged mice. Moreover, high-fat feeding is considered a chronic low-grade inflammatory state. In high-fat fed mice, overexpression of CTRP-3 reduced the proinflammatory cytokines such as serum IL-5 and TNF-*α* and elevated soluble gp130 (sgp130) levels [32]. Soluble gp130 is known to antagonize the inflammatory responses by binding to the cytokines of IL-6 family [33], and its serum levels are higher in older individuals with metabolic syndrome [34]. TNF-*α* is a potent inducer of insulin resistance [35], whereas IL-5 can activate the eosinophil cells to participate in inflammation [36]. In high-fat fed CTRP-3- transgenic mice, the attenuated systemic inflammation was accompanied with improved insulin sensitivity [14]. In our study, plasma IL-6 concentrations were significantly higher in subjects with pre-DM and nT2DM, and the multiple linear regression analyses also showed that IL-6 was an independent risk factor for CTRP-3. Though the causality cannot be concluded, our results suggest that CTRP-3 may participate in the pathogenesis of inflammation mediated diabetes.

Conventionally, HMGB-1 was considered as a nuclear protein to regulate gene transcription. It was not until 1999 that the cytosolic HMGB-1 was found to be a proinflammatory mediator and can be secreted by activated macrophages in the context of infection, injury, or other inflammatory status [37]. There are several pathways by which HMGB-1 can induce inflammation. First, HMGB-1 can upregulate the advanced glycation end-product (RAGE) signaling by binding to the RAGE receptor, thus boosting inflammation response [38]. Second, HMGB-1 can activate the nuclear factor-*κ*B (NF-*κ*B) in metabolic disease [21]. In cells, activated NF-*κ*B enables the biosynthesis of several proinflammatory mediators such as tumor necrosis factor-*α* (TNF-*α*), interleukin-6 (IL-6), and IL-1*β*, which participate in the pathological inflammatory response [39]. In addition, HMGB-1 can bind to the Toll-like receptor 2 (TLR2) and TLR4. As a classical innate immunity pathway, the TLRs can affect the development and progression of diabetes through the NF-*κ*B signaling [40]. Through MyD88 signaling, activated TLR2 and TLR4 can trigger the release of proinflammatory factors such as IL-6, TNF-*α*, and IL-1*β* and the aggregation of inflammatory cells [41]. Inflammatory microenvironment could cause *β*-cell dysfunction [42] and trigger insulin resistance, which in turn causes gradual progression to T2DM [43, 44]. As mentioned above, inflammation and insulin resistance already exist in prediabetic stage and are important contributors to the development of diabetes. In our study, we found the plasma HMGB-1 and IL-6 levels were gradually increased across the pre-DM and nT2DM group. Partial correlation and linear regression analyses showed a significant association among plasma HMGB-1, HOMA-IR, and IL-6. Further multiple logistical analyses also demonstrated that plasma HMGB-1 levels were associated with pre-DM and nT2DM after adjusting for several confounders. As reported by Schierbeck et al. [45], circulating HMGB-1 levels were increased in adipocytes from insulin-resistant subjects, while serum HMGB-1 could promote insulin release in INS-1 cells. In a recent study, Giacobbe et al. [46] reported that HMGB-1 was associated with the presence of gestational diabetes mellitus (GDM) and insulin resistance in the third trimester of pregnancy. Insulin resistance and inflammation are involved in the pathogenesis of GDM; women with GDM have higher risk to develop T2DM after pregnancy. Combined with above evidence, we speculate that the increased plasma HMGB-1 concentrations may largely be due to the presence of insulin resistance and the low chronic inflammation in the context of hyperglycemia.

Our study has some limitations. First, this cross-sectional study could not conclude the causality between plasma CTRP-3/HMGB-1 and pre-DM or diabetes; thus a prospective study is required in future research. Second, due to the small sample size in our study, it is important to determine the cut-off value of circulating CTRP-3 and HMGB-1 to predict pre-DM and T2DM in a large sample size including the sex, race, and other potential confounders. Besides, contrary to other studies, we found no significant difference in plasma CTRP-3 and HMGB-1 by gender; more researches are needed to clarify the influence by gender.

In conclusion, we found plasma CTRP-3 levels were lower whereas plasma HMGB-1 concentrations were higher in subjects with prediabetes and newly diagnosed T2DM. Circulating CTRP-3 and HMGB-1 concentrations might be promising biomarkers to predict prediabetes and T2DM.

## Supplementary Material

Supplemental Fig. 1 The flow chart of the inclusion and exclusion criteria.

## Figures and Tables

**Table 1:** Clinical and biochemical characteristics of the study subjects.

Variables	NGT	Pre-DM	nT2DM	*F* value	*P* value
Participants (M/F)	62 (30/32)	111 (55/56)	56 (27/29)		
Age (years)	52.7 ± 5.6	53.6 ± 5.5	56.6 ± 8.05^*∗*†^	6.399	0.002
FPG (mmol/L)	5.0 ± 0.3	6.4 ± 0.3^*∗*^	7.9 ± 0.7^*∗*†^	72.24	<0.001
INS	5.5 ± 0.9	5.9 ± 0.9^*∗*^	7.3 ± 1.2^*∗*†^	37.63	<0.001
HOMA-IR	1.09 ± 0.17	1.71 ± 0.11^*∗*^	2.69 ± 0.39^*∗*†^	52.08	<0.001
2 h PG (mmol/L)	6.8 ± 0.6	9.3 ± 1.3^*∗*^	13.1 ± 1.6^*∗*†^	25.17	<0.001
HbA1c (%)	4.9 ± 0.6	6 ± 0.2^*∗*^	7.6 ± 0.6^*∗*†^	63.21	<0.001
BMI (kg/m^2^)	22.42 ± 1.78	22.97 ± 2.04	23.84 ± 2.05^*∗*†^	7.663	0.001
WHR	0.83 ± 0.06	0.85 ± 0.05^*∗*^	0.88 ± 0.03^*∗*†^	21.486	<0.001
SBP (mmHg)	128 ± 13	129 ± 14	132 ± 12	1.19	0.306
DBP (mmHg)	77 ± 7	77 ± 10	79 ± 9	2.268	0.106
TC (mmol/L)	4.51 ± 0.6	4.54 ± 0.72	4.77 ± 0.61^*∗*†^	2.85	0.06
TG (mmol/L)	1.12 ± 0.5	1.36 ± 0.65^*∗*^	1.6 ± 0.79^*∗*†^	7.968	<0.001
LDL-C (mmol/L)^#^	0.77 ± 0.26	0.84 ± 0.28	0.92 ± 0.16^*∗*†^	5.182	0.006
HDL-C (mmol/L)	1.4 ± 0.34	1.24 ± 0.28^*∗*^	1.04 ± 2.06^*∗*†^	12.163	<0.001
ALT (U/L)	20 ± 6	20 ± 8	23 ± 10	1.68	0.189
AST (U/L)	22 ± 8	23 ± 7	24 ± 7^*∗*^	2.562	0.079
*γ*-GGT (U/L)^#^	2.78 ± 0.38	3.07 ± 0.55^*∗*^	3.42 ± 0.65^*∗*†^	21.01	<0.001
Cr	63 ± 8	63 ± 14	64 ± 8	0.397	0.673
IL-6 (pg/mL)	47.4 ± 7.1	59.44 ± 6.79^*∗*^	77.58 ± 4.03^*∗*†^	39.46	<0.001

Data were expressed as means ± standard deviation. Nonnormally distributed parameters including LDL-C and *γ*-GGT were logarithmically transformed before analyses. Differences between multiple groups were tested by analysis of variance (ANOVA) for continuous variables. NGT, normal glucose tolerance; pre-DM, prediabetes states (including IFG, impaired fasting glucose; IGT, impaired glucose tolerance; IGR, impaired glucose regulation); T2DM, type 2 diabetes mellitus; M, male; F, female; FPG, fasting plasma glucose; FINS, fasting serum insulin; HOMA-IR, homeostasis model assessment for insulin resistance; 2 h PG, 2 h postchallenge plasma glucose; HbA1c, hemoglobin A1c; BMI, body mass index; WHR, waist-to-hip ratio; SBP, systolic blood pressure; DBP, diastolic blood pressure; TC, total cholesterol; TG, triglyceride; LDL-C, low-density lipoprotein cholesterol; HDL-C, high-density lipoprotein cholesterol; AST, aspartate transaminase; ALT, alanine transaminase; *γ*-GGT, gamma-glutamyl transpeptidase; IL-6, interleukin-6.

^#^Logarithmically transformed variables.

^*∗*^ *P* < 0.05 compared with NGT, ^†^ *P* < 0.05 compared with pre-DM.

**Table 2:** Spearman and partial correlation analyses between variables and plasma CTRP-3 and HMGB-1.

	Plasma CTRP-3		Plasma CTRP-3 (age- and sex-adjusted)		Plasma HMGB-1		Plasma HMGB-1 (age- and sex-adjusted)	
	*r*	*P*	*r*	*P*	*r*	*P*	*r*	*P*
Age (years)	−0.152	0.021			0.106	0.111		
FPG (mmol/L)	−0.745	<0.001	−0.756	<0.001	0.615	<0.001	0.61	<0.001
INS	−0.635	<0.001	−0.626	<0.001	0.604	<0.001	0.529	<0.001
HOMA-IR	−0.763	<0.001	−0.756	<0.001	0.693	<0.001	0.689	<0.001
2 h PG (mmol/L)	−0.67	<0.001	−0.66	<0.001	0.61	<0.001	0.604	<0.001
HbA1c (%)	−0.806	<0.001	−0.802	<0.001	0.677	<0.001	0.674	<0.001
BMI (kg/m^2^)	−0.219	0.001	−0.202	0.002	0.216	0.001	0.203	0.002
WHR	−0.374	<0.001	−0.357	<0.001	0.255	<0.001	0.242	<0.001
SBP (mmHg)	−0.089	0.181	−0.085	0.202	0.129	0.052	0.124	0.062
DBP (mmHg)	−0.14	0.034	−0.127	0.054	0.124	0.061	0.087	0.108
TC (mmol/L)	−0.094	0.154	−0.095	0.156	0.033	0.624	0.032	0.145
TG (mmol/L)	−0.213	0.001	−0.188	0.004	0.163	0.014	0.145	0.029
LDL-C (mmol/L)	−0.152	0.021	−0.142	0.032	0.25	<0.001	0.243	<0.001
HDL-C (mmol/L)	0.369	<0.001	0.372	<0.001	−0.403	<0.001	−0.405	<0.001
ALT (U/L)	−0.02	0.769	−0.014	0.829	0.022	0.736	0.02	0.77
AST (U/L)	−0.07	0.292	−0.064	0.342	0.024	0.722	0.019	0.772
*γ*-GGT (U/L)	−0.124	0.032	−0.115	0.051	0.136	0.027	0.085	0.114
Cr	−0.056	0.4	−0.055	0.41	0.066	0.324	0.067	0.319
IL-6 (pg/mL)	−0.797	<0.001	−0.781	<0.001	0.696	<0.001	0.695	<0.001

Correlations between variables were analyzed by Spearman's correlation test and age- and sex-adjusted partial correlation test. CTRP-3, C1q/TNF-related protein-3; HMGB-1, high-mobility group box-1; FPG, fasting plasma glucose; FINS, fasting serum insulin; HOMA-IR, homeostasis model assessment for insulin resistance; 2 h PG, 2 h postchallenge plasma glucose; HbA1c, hemoglobin A1c; BMI, body mass index; WHR, waist-to-hip ratio; SBP, systolic blood pressure; DBP, diastolic blood pressure; TC, total cholesterol; TG, triglyceride; LDL-C, low-density lipoprotein cholesterol; HDL-C, high-density lipoprotein cholesterol; AST, aspartate transaminase; ALT, alanine transaminase; *γ*-GGT, gamma-glutamyl transpeptidase; IL-6, interleukin-6.

**Table 3:** Multiple logistic regression analyses of plasma CTRP-3 levels among various groups.

	Pre-DM		nT2DM	
	OR, 95% CI	*P* value	OR, 95% CI	*P* value
Age, sex	0.963 (0.952, 0.974)	<0.001	0.934 (0.92, 0.949)	<0.001
+ALT, AST, GGT, Cr	0.963 (0.952, 0.975)	<0.001	0.934 (0.919, 0.949)	<0.001
+SBP, DBP	0.961 (0.949, 0.973)	<0.001	0.931 (0.916, 0.947)	<0.001
+TC, TG, LDL-C, HDL-C	0.96 (0.947, 0.973)	<0.001	0.931 (0.915, 0.949)	<0.001
+WHR, BMI	0.961 (0.948, 0.974)	<0.001	0.933 (0.916, 0.951)	<0.001

Pre-DM, prediabetes states (including IFG, impaired fasting glucose; IGT, impaired glucose tolerance; IGR, impaired glucose regulation); T2DM, type 2 diabetes mellitus; OR, odds ratio; CI, confidence interval; ALT, alanine transaminase; AST, aspartate transaminase; *γ*-GGT, gamma-glutamyl transpeptidase; Cr, creatinine; SBP, systolic blood pressure; DBP, diastolic blood pressure; TC, total cholesterol; TG, triglyceride; LDL-C, low-density lipoprotein cholesterol; HDL-C, high-density lipoprotein cholesterol; WHR, waist-to-hip ratio; BMI, body mass index.

**Table 4:** Multiple logistic regression analyses of plasma HMGB-1 levels among various groups.

	Pre-DM		nT2DM	
	OR, 95% CI	*P* value	OR, 95% CI	*P* value
Age, sex	5.436 (2.492, 11.858)	<0.001	14.436 (6.899, 26.796)	<0.001
+ALT, AST, GGT, Cr	5.142 (2.286, 11.564)	<0.001	9.81 (3.509, 15.284)	<0.001
SBP, DBP	5.173 (2.288, 11.696)	<0.001	10.798 (4.641, 21.068)	<0.001
TC, TG, LDL-C, HDL-C	5.505 (2.183, 13.879)	<0.001	7.805 (2.163, 17.853)	<0.001
WHR, BMI	5.115 (1.993, 13.125)	<0.001	3.754 (1.204, 9.281)	<0.001

Pre-DM, prediabetes states (including IFG, impaired fasting glucose; IGT, impaired glucose tolerance; IGR, impaired glucose regulation); T2DM, type 2 diabetes mellitus; OR, odds ratio; CI, confidence interval; ALT, alanine transaminase; AST, aspartate transaminase; *γ*-GGT, gamma-glutamyl transpeptidase; Cr, creatinine; SBP, systolic blood pressure; DBP, diastolic blood pressure; TC, total cholesterol; TG, triglyceride; LDL-C, low-density lipoprotein cholesterol; HDL-C, high-density lipoprotein cholesterol; WHR, waist-to-hip ratio; BMI, body mass index.
