# Supplementary figures and images for: Plasma C1q/TNF-Related Protein-3 (CTRP-3) and High-Mobility Group Box-1 (HMGB-1) Concentrations in Subjects with Prediabetes and Type 2 Diabetes
Source: J Diabetes Res. 2016 Sep 22;2016:9438760. doi: 10.1155/2016/9438760 (PMC5055961; doi:10.1155/2016/9438760)

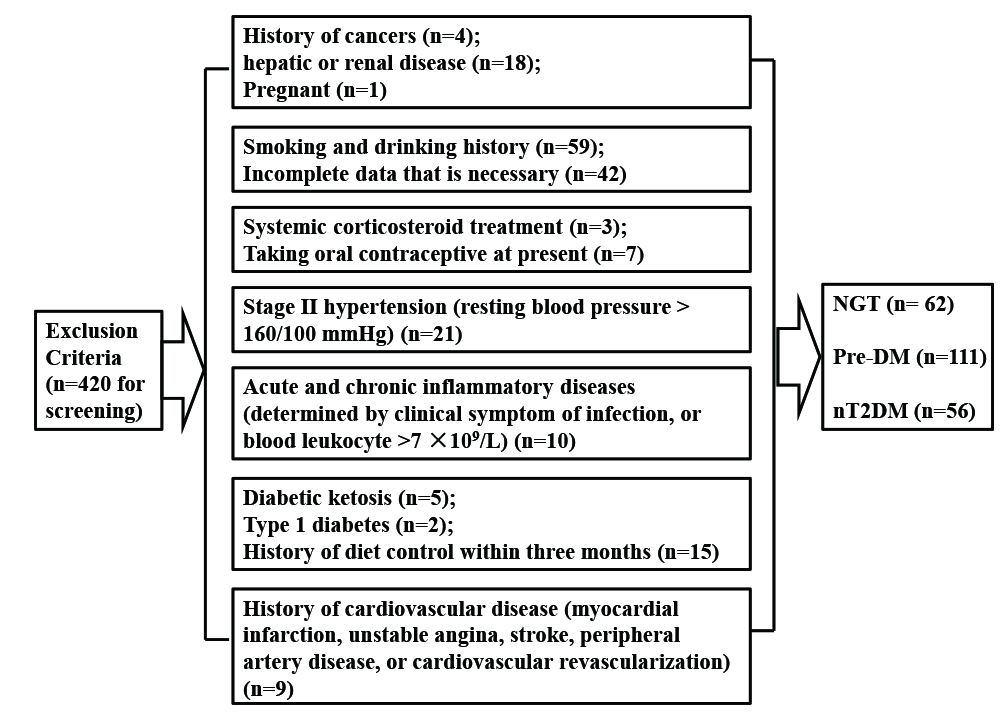

Supplement: Supplementary file 1 — Supplemental Fig. 1 The flow chart of the inclusion and exclusion criteria. [file 9438760.f1.tif]
